# Superior high creep resistance of *in situ* nano-sized TiC_x_/Al-Cu-Mg composite

**DOI:** 10.1038/s41598-017-04816-0

**Published:** 2017-07-03

**Authors:** Lei Wang, Feng Qiu, Qinglong Zhao, Min Zha, Qichuan Jiang

**Affiliations:** 1 State Key Laboratory of Automotive Simulation and Control, Jilin university, No. 5988 Renmin Street, Changchun, 130025 PR China; 2 Key Laboratory of Automobile Materials, Ministry of Education, and Department of Materials Science and Engineering, Jilin University, No. 5988 Renmin Street, Changchun, 130025 PR China

## Abstract

The tensile creep behavior of Al-Cu-Mg alloy and its composite containing *in situ* nano-sized TiC_x_ were explored at temperatures of 493 K, 533 K and 573 K with the applied stresses in the range of 40 to 100 MPa. The composite reinforced by nano-sized TiC_x_ particles exhibited excellent creep resistance ability, which was about 4–15 times higher than those of the unreinforced matrix alloy. The stress exponent of 5 was noticed for both Al-Cu-Mg alloy and its composite, which suggested that their creep behavior was related to dislocation climb mechanism. During deformation at elevated temperatures, the enhanced creep resistance of the composite was mainly attributed to two aspects: (a) Orowan strengthening and grain boundary (GB) strengthening induced by nano-sized TiC_x_ particles, (b) *θ*′ and *S*′ precipitates strengthening.

## Introduction

Elevated-temperature creep properties of particles-reinforced Al matrix composites (AMCs) have been comprehensively investigated during the last decades, primarily due to their high potential as light weight structural materials under elevated-temperature^1–12^. Particles such as Al_2_O_3_^3^, SiC^1, 2, 6–10^, TiC^11^ and B_4_C^4^ introduced to composites generally act as barriers to hinder dislocation motion and lead to an enhanced creep resistance. Nevertheless, various particle sizes have remarkable effects on the creep properties of particles-reinforced AMCs. For instance, Tjong *et al*.^6^ pointed out that the composites reinforced with fine SiC_p_ particles (3.5 µm) had much lower creep rate than pure Al, while that for the composites reinforced with large SiC particles (10 and 20 µm) was almost identical to pure Al. Similar. results were also obtained by Pandey *et al*.^12^. Hence, research on the creep behavior of fine-sized particles, especially nano-sized particles reinforced AMCs has become significant importance.

Previous studies primarily focused on the correlation between nano-sized particles dispersion and mechanical properties of AMCs containing nano-sized particles, which was known to depend on the fabrication methods and their parameters^13, 14^. Commonly the nano-sized particles can be distributed in the Al matrix by *ex situ* and *in situ* fabrication methods. For example, a study on creep behaviors of Al-Al_2_O_3_ nanocomposite prepared by *ex situ* method of mechanical milling and hot powder extrusion has been carried out by Monazzah *et al*.^15^ at temperatures of 648K-723K. They revealed that the nano-sized Al_2_O_3_ particles actively pinned the substructure and limited the deformation of Al matrix and hence increased the creep resentence. Also, Lin *et al*.^2^ introduced nano-sized Al_2_O_3_ particles into 2014 Al alloy by powder metallurgy (PM). The interaction between nano-sized particles and moving dislocations indicated that the presence of nano-sized Al_2_O_3_ particles in the 2014 Al alloy was responsible for the threshold stress of creep. However, the reinforced particles added into Al matrix by traditional *ex situ* methods are easily to be polluted. Alternatively, *in situ* fabrication methods offer advantages of cleaner particle-matrix interface and can meet the low energy requirement, which have been considered by many researchers^13, 16, 17^. Detailed research on the creep behavior of TiC_*p*_ (average size of 0.5–1.5 μm)/2618Al composites prepared by an *in situ* combustion synthesis method has been reported by Ji *et al*.^11^. The results revealed an increased creep resistance (about 2–6 times higher) of the composites comparing to the 2618Al alloy within the studying temperatures of 523K-623k. However, little work has been performed on the creep behavior of *in situ* fabricated nano-sized particles reinforced AMCs.

To our knowledge, studies on the creep resistance of nano-sized particles reinforced AMCs prepared by combustion synthesis method have not been discussed in literature. In previous studies, Al-Cu-Mg composites containing nano-sized TiC_x_ particles prepared by the present *in situ* method assisted with hot pressure, which possessed excellent tensile properties at elevated temperatures due to the presence of nano-sized TiC_x_ particles, have been studied^18, 19^. Accordingly, the current research was to comparatively investigate the creep behavior of Al-Cu-Mg composite reinforced by *in situ* nano-sized TiC_x_ particles and its matrix alloy at elevated temperatures. Effects of nano-sized TiC_x_ particles on creep behavior of the composite and enhancement mechanisms were investigated at 493 K, 533 K and 573 K. Our results will provide a practical approach to prepare Al matrix composites with high creep resistance.

## Results

### Microstructural characterization of the nano-sized TiC_x_/Al-Cu-Mg composite and its matrix

**Figure 1:**
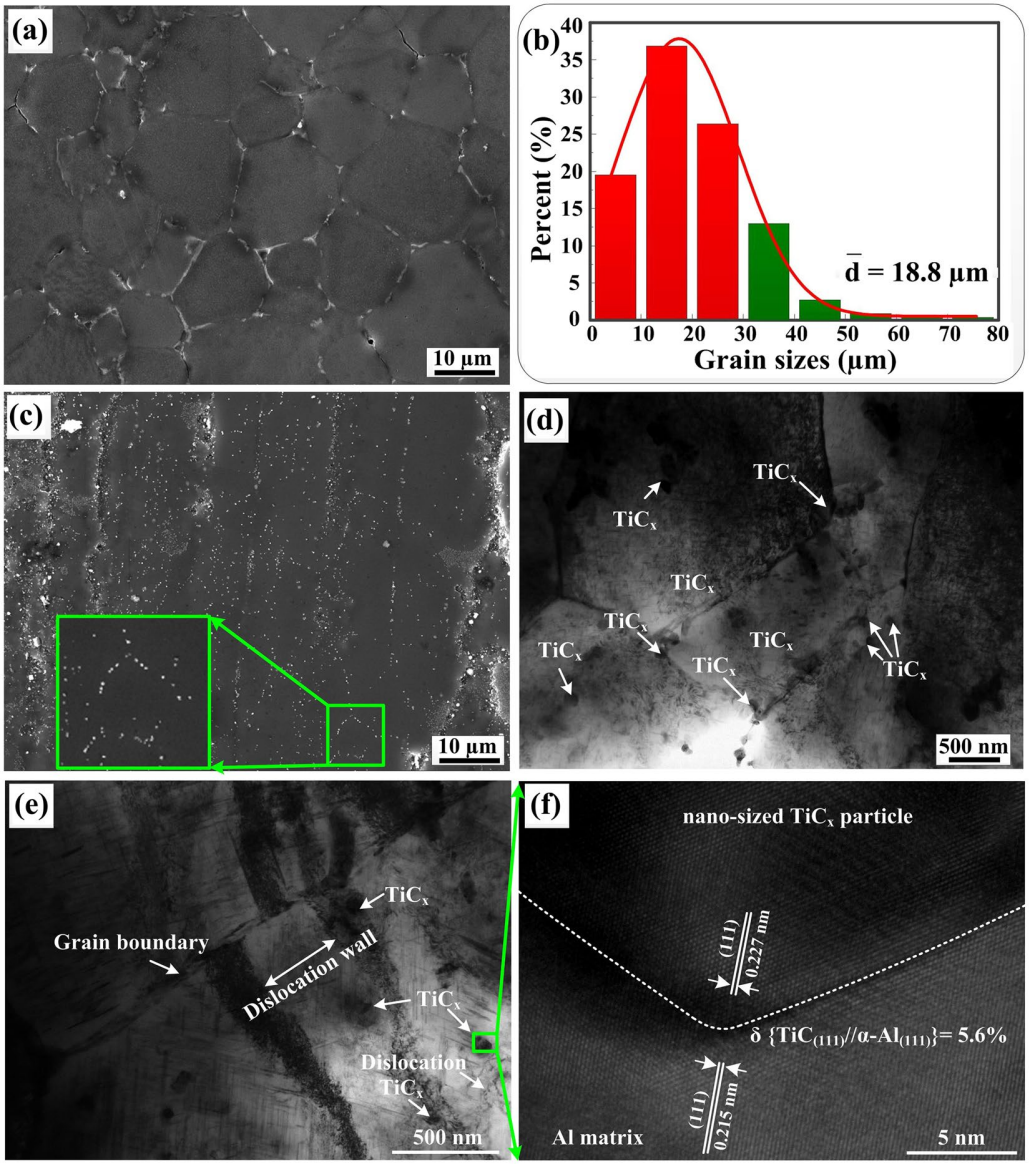
(**a**) and (**c**) SEM micrographs of as-extruded Al-Cu-Mg alloy and nano-sized TiC_x_/Al-Cu-Mg composite after T4 treatment; (**b**) Size distribution of the fully recrystallized grains for Al-Cu-Mg alloy; (**d**) TEM images of the distribution of nano-sized TiC_x_ particles in composite; (**e**) TEM images of the dislocations and nano-sized TiC_x_ particles in composite; (**f**) High resolution image for the interface of nano-sized TiC_x_ particles and Al matrix.

Figure 1a and c shows SEM micrographs of as-extruded Al-Cu-Mg alloy and its composite reinforced by nano-sized TiC_x_ after solution-treating at 783 K for 60 min, water quenching and followed by aging at room temperature for 96 h (T4 treatment). As indicated, a fully recrystallized microstructure is observed in Fig. 1a and the mean grain size of the unreinforced alloy is about 18.8 µm (Fig. 1b). But recrystallization of the present composite seems to be retarded due to the presence of nano-sized TiC_x_ particles, as shown in Fig. 1c. Figure 1d exhibits TEM images of the distribution of nano-sized TiC_x_ particles in composite. It can be viewed that nano-sized TiC_x_ particles are located both at grain boundaries and in grain interiors. In addition, the composite contains a mass of dislocations before creep deformation (Fig. 1e). Figure 1f shows a high resolution image for the interface between nano-sized TiC_x_ particles and Al matrix, which displays a good particle-Al matrix interfacial bonding. Moreover, a lattice mismatch of 5.6% between nano-sized TiC_x_ particles and Al matrix is observed.

**Figure 2:**
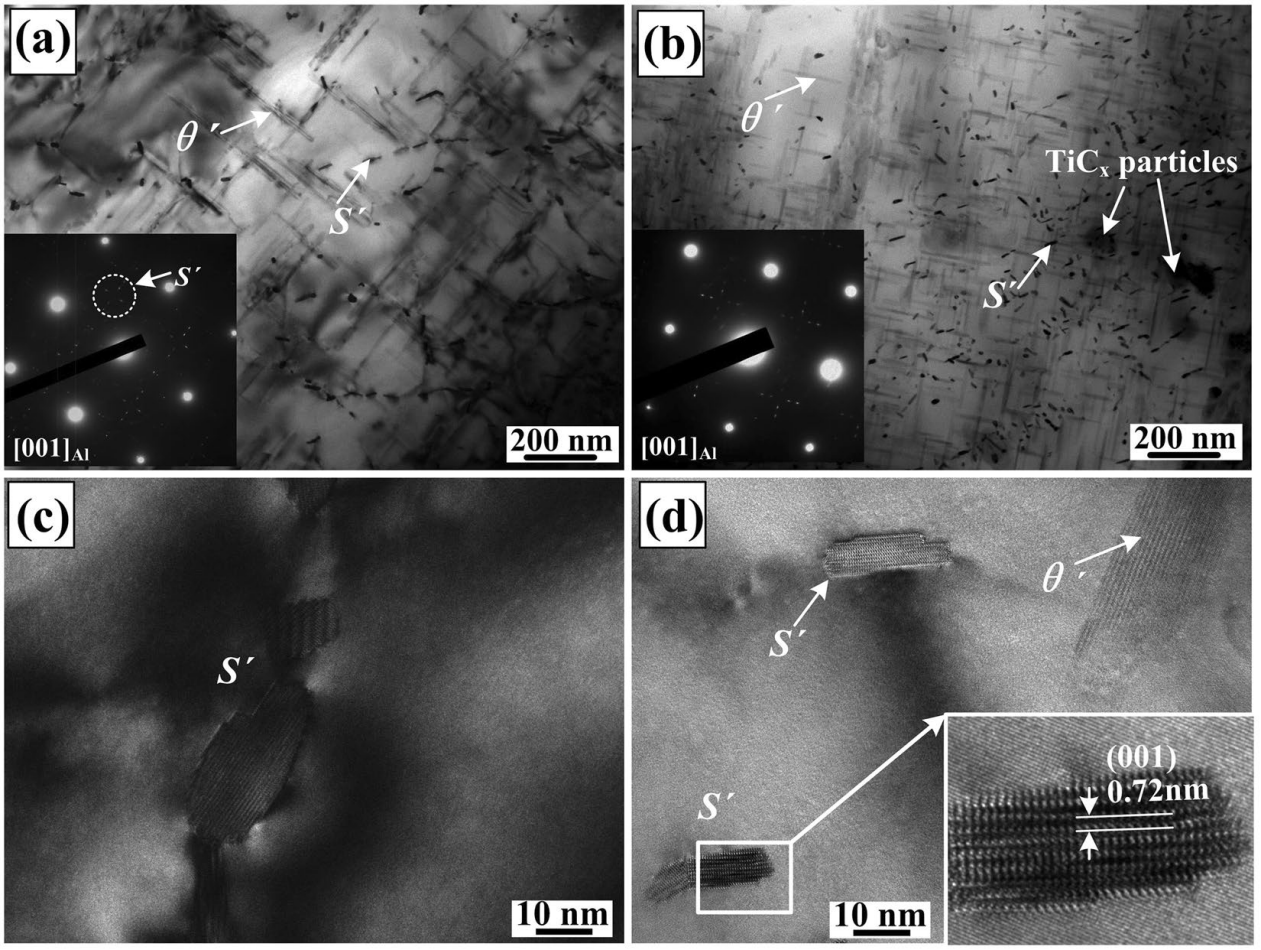
TEM micrographs of *θ*′ and *S*′ precipitates in (**a**) Al-Cu-Mg alloy and (**b**) nano-sized TiC_x_/Al-Cu-Mg composites, insert: SAED pattern of α-Al from [001]_α-Al_ direction; HREM images of *S*′ precipitates for (**c**) Al-Cu-Mg alloy and (**d**) the composite.

Figure 2a,b illustrate TEM images and corresponding selected area electron diffraction (SAED) patterns of the samples for Al-Cu-Mg alloy and the composite containing 9 vol.% nano-sized TiC_x_ particles. As indicated, *θ*′ precipitates in the composite (Fig. 2b) have a smaller average length and larger number density as compared to those in the Al-Cu-Mg alloy (Fig. 2a). The mean length of *θ*′ precipitates in the composite is about 80 nm, while that in the Al-Cu-Mg alloy is about 150 nm. This is mainly due to the small-sized recrystallized grains and retarded recrystallization in the present composite induced by the nano-sized TiC_x_ particles^20^. Moreover, *S*′ precipitates, which is confirmed by high resolution image in Fig. 2d, show an inhomogeneous distribution in both Al-Cu-Mg alloy and its composite. In the meantime, the number density of *S*′ precipitates remarkably increases accompanying a decrease in average size. It has been reported that dislocations can act as the sites for heterogeneous nucleation of *S*′ precipitates during aging process^21, 22^. Dislocations usually generate in the composite by quenching and a coefficient of thermal expansion (CTE) mismatch between nano-sized TiC_x_ particles and the matrix. Hence, *S*′ precipitates tend to precipitate out around nano-sized TiC_x_ particles, as shown in Fig. 2b. Meanwhile, compared with Al-Cu-Mg alloy, an increase in dislocation density indicates that the heterogeneous nucleation sites for *S*′ precipitates increase in the composites, resulting in a decrease in precipitate size, as shown in Fig. 2c,d.

### Creep behavior analysis

**Figure 3:**
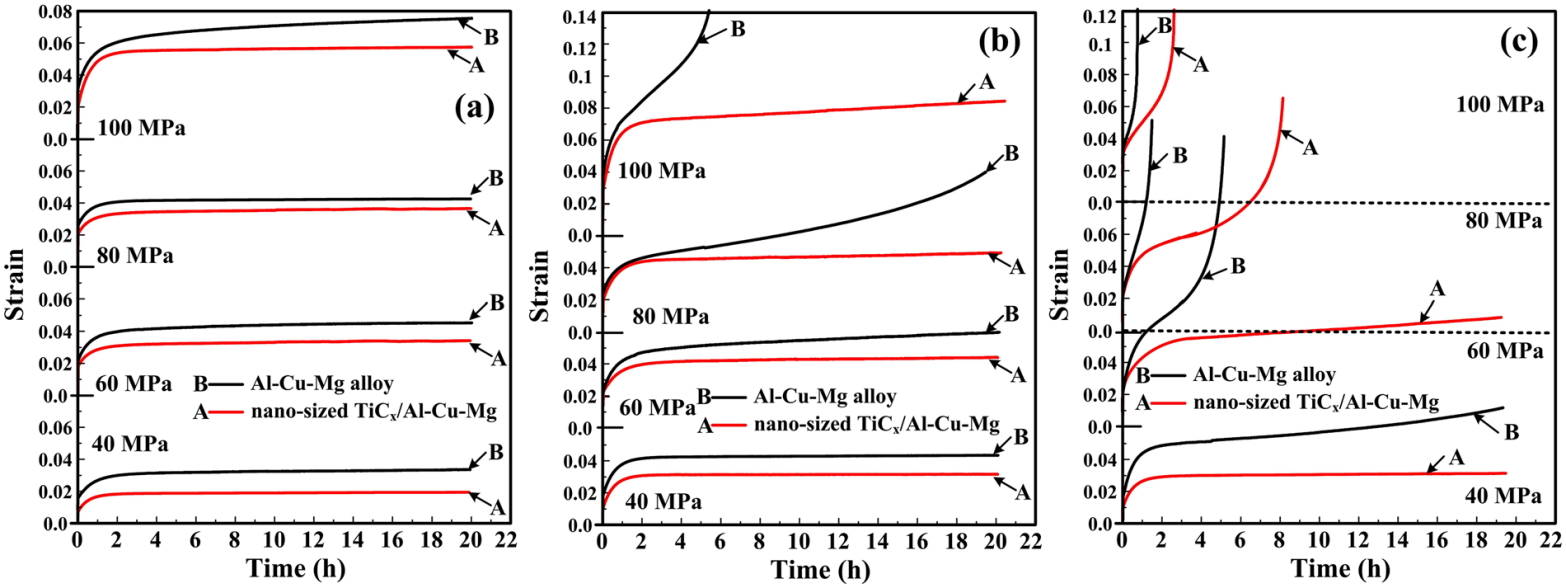
Creep curves of Al-Cu-Mg alloy and nano-sized TiC_x_/Al-Cu-Mg composite obtained with applied stress of 40 MPa, 60 MPa, 80 MPa and 100 MPa at (**a**) 493 K, (**b**) 533 K and (**c**) 573 K.

**Figure 4:**
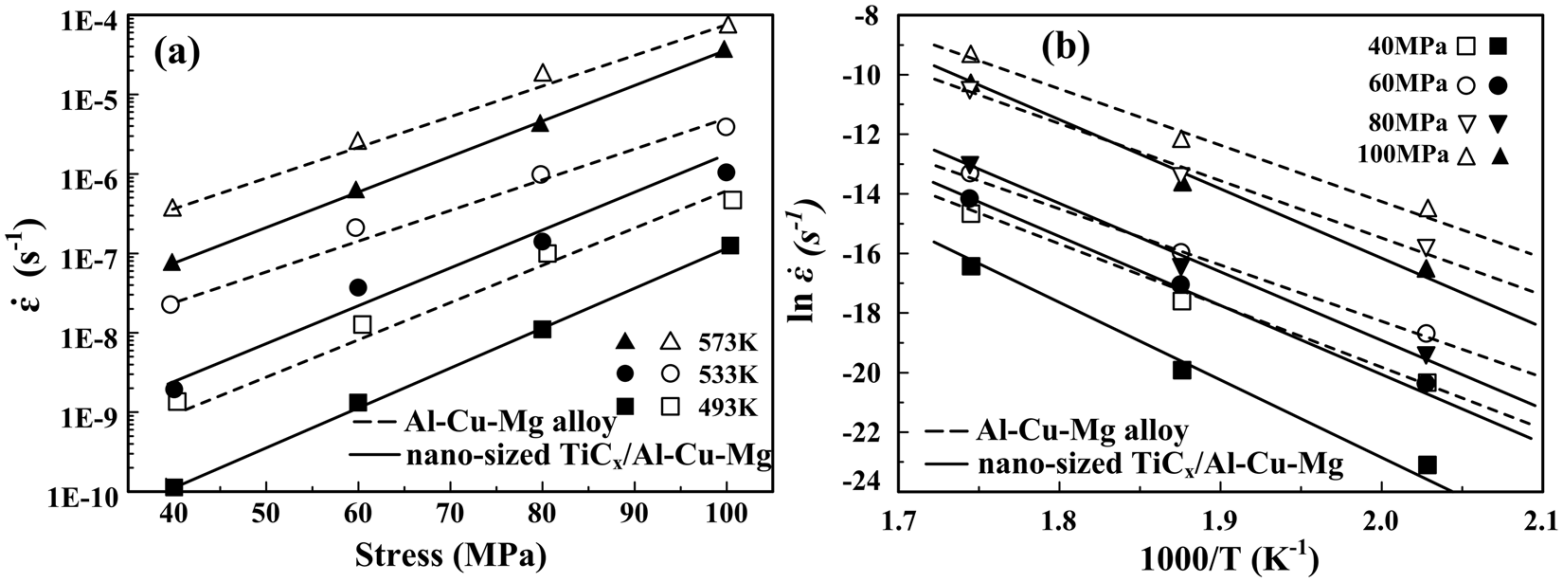
(**a**) Variation of ${\dot{\varepsilon }}_{m}$ with the applied stress (σ) for Al-Cu-Mg alloy and the composite at 493 K, 533 K and 573 K; (**b**) the plots of $\mathrm{ln}\,\dot{\varepsilon }$ versus 1000/T for the Al-Cu-Mg alloy and the composite at various applied stress.

Creep curves (strain *vs*. creep time) for the samples of Al-Cu-Mg alloy and its composite obtained with applied stress of 40 MPa, 60 MPa, 80 MPa and 100 MPa at (a) 493 K, (b) 533 K and (c) 573 K are showed in Fig. 3. At 493 K, each of these creep curves includes primary creep strain and steady-state creep strain. At 533 K, with the increase of the applied stress, creep curve of nano-sized TiC_x_/Al-Cu-Mg composite is characterized by a long primary stage followed by a very long secondary stage. In contrast, the tertiary stage appears for the Al-Cu-Mg alloy. At 573 K, with increasing applied stress, the creep rate reaches a steady state in secondary stage and accelerates in the tertiary stage for both Al-Cu-Mg alloy and its composite, leading to fracture. Figure 4a indicates the variation of the steady-state creep rate ($\dot{\varepsilon }$) with the applied stress (σ) for Al-Cu-Mg alloy and the composite containing nano-sized TiC_x_ at 493 K, 533 K and 573 K. All the steady-state creep rate data for Al-Cu-Mg alloy and the composite under applied stresses of 40–100 MPa at temperatures of 493–573 K are showed in Table 1. The minimum creep rates of the composite (1.47 × 10^−9^~8.52 × 10^−5^) are almost 4–15 times lower than those of Al-Cu-Mg alloy (1.01 × 10^−10^~1.54 × 10^−5^) under the same creep conditions. For example, the minimum creep rate of the composite is about 15 times lower than that of Al-Cu-Mg alloy under the applied stress of 40 MPa at 493 K. Similarly, the minimum creep rate value for the composite is about 4 times lower than that for Al-Cu-Mg alloy under 100 MPa at 533 K. Clearly, creep resistance of the composite is actually enhanced due to the presence of nano-sized TiC_x_ particles.

**Table 1:** The steady-state creep rate data of Al-Cu-Mg alloy and 9 vol.% nano-sized TiC_x_/Al-Cu-Mg composite under different applied stresses of 40, 60, 80 and 100 MPa at various temperatures

Stress (MPa)	493 K		533 K		573 K	
	Al-Cu-Mg	composite	Al-Cu-Mg	composite	Al-Cu-Mg	composite
40	1.47 × 10^−9^	1.01 × 10^−10^	2.25 × 10^−8^	1.74 × 10^−9^	4.28 × 10^−7^	7.16 × 10^−8^
60	1.36 × 10^−8^	1.18 × 10^−9^	2.08 × 10^−7^	3.31 × 10^−8^	2.96 × 10^−6^	5.86 × 10^−7^
80	1.06 × 10^−7^	9.87 × 10^−9^	9.67 × 10^−7^	1.26 × 10^−7^	2.12 × 10^−5^	4.01 × 10^−6^
100	4.96 × 10^−7^	1.03 × 10^−7^	3.81 × 10^−6^	9.35 × 10^−7^	8.52 × 10^−5^	1.54 × 10^−5^

Furthermore, linear relationship between $\dot{\varepsilon }$ with the applied stress at each temperature is also observed in Fig. 4a, indicating that both the present composite and Al-Cu-Mg alloy follow the power law relationship. The power law creep equation can be given as:^23^

1 $$\dot{\varepsilon }=A\,{(\sigma )}^{n}\exp (-\frac{{Q}_{a}}{RT})$$

where $\dot{\varepsilon }$, *A*, *σ*, *n*, *Q*_*a*_, *R* and *T* represent the minimum creep rate, the structure dependent constant, the applied stress, the apparent stress exponent, the apparent activation energy, the gas constant and the absolute temperature, respectively. According to Eq. (1), when the temperature increases, the creep rates increase as well. Under the same tested temperature, the creep rates for both Al-Cu-Mg alloy and the composite increase when the applied stress increases. The apparent stress exponent values (*n-*values) can be calculated from the slopes of straight lines by relating $\mathrm{log}\,\dot{\varepsilon }$ and $\mathrm{log}\,\sigma$. The *n-*values are 8.3, 7.1 and 6.6 for the composite at 493 K, 533 K and 573 K, respectively, which are much higher than the *n-*values of 6.4, 5.6 and 5.8 for Al-Cu-Mg alloy. Figure 4b presents the plots of $\mathrm{ln}\,\dot{\varepsilon }$ versus 1000/T for the Al-Cu-Mg alloy and the composite at various applied stress. According to the formula (2): ${Q}_{a}$ *=* *−R* $\frac{\partial \,\mathrm{ln}\,\dot{\varepsilon }}{\partial (1000/T)}$, the apparent activation energy values are calculated as 215, 210, 204, 200 kJ/mol for the composite and 177, 163, 155, 150 kJ/mol for Al-Cu-Mg alloy at 40, 60, 80 and 100 MPa, respectively. Apparently, both the apparent stress exponent and apparent activation energy for the composites display higher values in comparison to the unreinforced matrix alloy.

## Discussion

**Figure 5:**
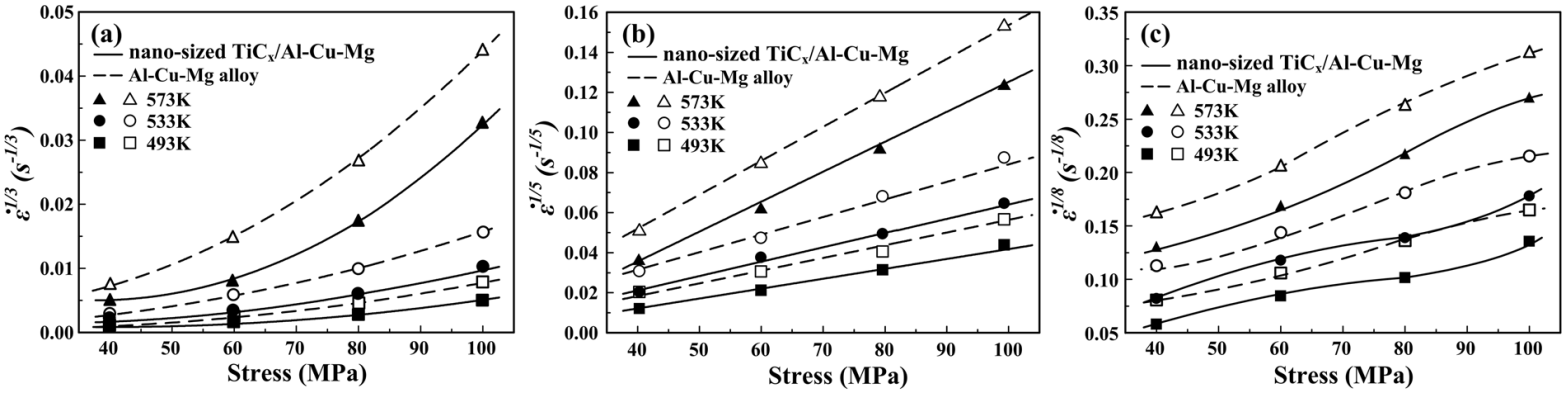
Lagneborg-Bergman plots of ${\dot{\varepsilon }}^{1/n}$ vs. stress for n = (**a**) 3, (**b**) 5 and (**c**) 8 for Al-Cu-Mg alloy and the composite, respectively.

In order to describe the related deformation mechanisms during creep tests, Lagneborg-Bergman plot, i.e. ${\dot{\varepsilon }}^{1/{n}_{0}}$ versus the applied stresses on linear scales, is often used^24^. Commonly, the true stress exponent (*n*_0_) have values of 3, 5 and 8 for Al and AMCs, which respectively represent creep mechanisms including dislocation glide, dislocation climb and invariant substructure model^24–26^. Lagneborg-Bergman plots for the composite and unreinforced matrix alloy are showed in Fig. 5. The best linear fit to the data points is observed when the value of *n*_0_ is selected as 5, indicating that the creep behavior of both the composite and Al-Cu-Mg alloy is detected by dislocation climb mechanism. Note that various investigators have put forward different viewpoints on the value of *n*_0_. Hosseini Monazzah *et al*.^15^ pointed out that the value of *n*_0_ for hot extruded nano-sized Al_2_O_3_/Al composites decreased from 8 to 3 by increasing testing temperatures from 648 K-723 K. However, Mohamed *et al*.^2^ reported that *n*_0_-value of 5vol.% SiC (~100 nm)/2124Al composite (T4-treatment) increased from 3 to 5 with temperature increasing from 618 K-678 K., Moreover, the stress exponent of 5 obtained for the present composite is identical to that reported by Ji *et al*.^11^ on the 0.5–1.5 μm TiC_*p*_/2618Al composites and Liao *et al*.^10^ on the 15% micro-sized SiC_p_/AlLi composites.

**Figure 6:**
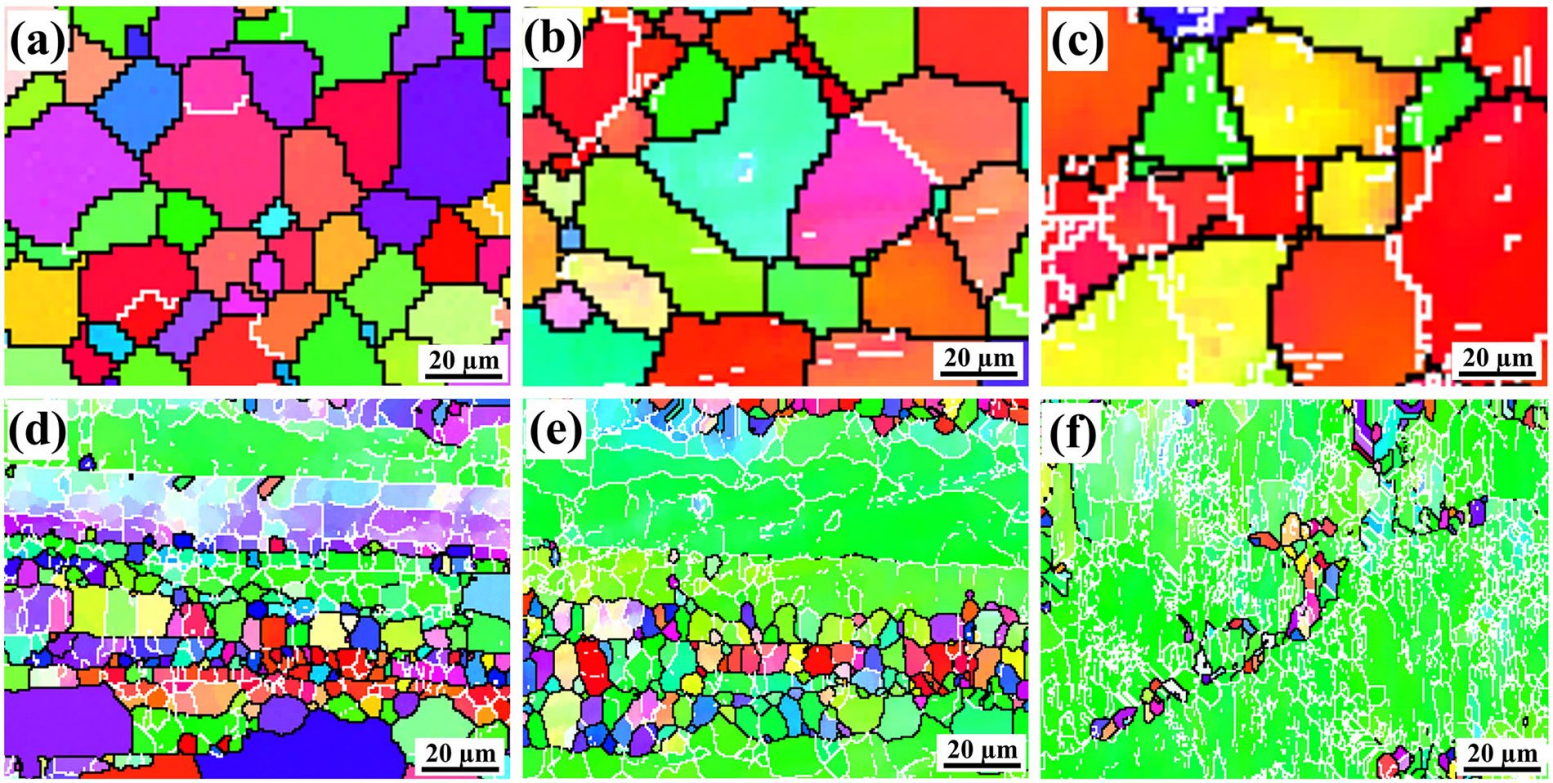
(**a**) and (**d**) Inverse pole figure (IPF) images of Al-Cu-Mg alloy and its composite before creep test respectively; (**b**) and (**e**) IPF images for Al-Cu-Mg alloy and its composite after testing at 493 K with 60 MPa; (**c**) and (**f**) IPF images for Al-Cu-Mg alloy and its composite after testing at 573 K with 60 MPa.

**Figure 7:**
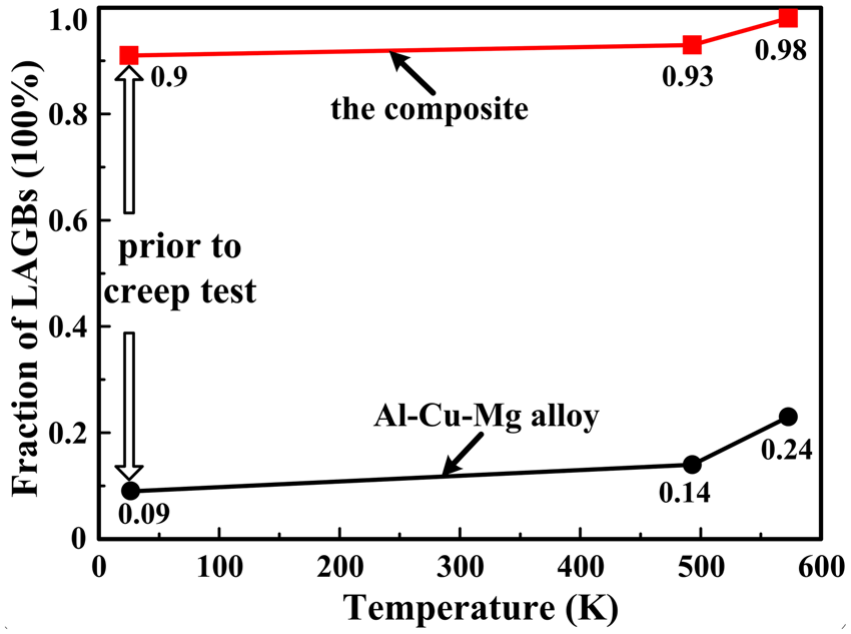
Variation of the fraction of LAGBs with the temperature for Al-Cu-Mg alloy and the composite.

Figure 6a and d shows inverse pole figure (IPF) maps for PM Al-Cu-Mg alloy and the present composite before creep tests. Figure 6b and e displays IPF images for Al-Cu-Mg alloy and its composite after testing at 493 K with 60 MPa, meanwhile IPF images for those samples tested at 573 K with 60 MPa are exhibited in Fig. 6c and f. The white and black lines observed from the EBSD maps respectively indicate the location of low angle grain boundaries (LAGBs, 2–15°) and high angle grain boundaries (HAGBs, ≥15°). Before creep tests, the sample for Al-Cu-Mg alloy exhibits a full recrystallized microstructure with grain size of ~18.8 µm, while the recrystallization of the composite is inhibited by nano-sized TiC_x_ particles. In the meantime, the microstructure for the composite consists of a very high fraction of LAGBs as compared to the Al-Cu-Mg alloy, i.e. 90% *vs*. 9%. Those mentioned above have been reported in our former study^19^. After tested at 493 K and 573 K, the mean grain sizes of Al-Cu-Mg alloy increase from 18.8 to 22.1 µm and 18.8 to 27.2 µm, respectively, as shown in Fig. 6b and c. But the mean grain sizes for the composite have no obvious change with testing temperature increases from 493 K to 573 K (Fig. 6e and f). Figure 7 shows variation of the fraction of LAGBs with the temperature for Al-Cu-Mg alloy and its composite. Clearly, the fraction of LAGBs for both Al-Cu-Mg alloy and the composite apparently increase with the increasing testing temperature. After tested at 493 K and 573 K, the fraction of LAGBs for Al-Cu-Mg alloy increases from 9% to 14% and 9% to 24%, while that for the composite increases from 90% to 93% and 90% to 98%, respectively.

**Figure 8:**
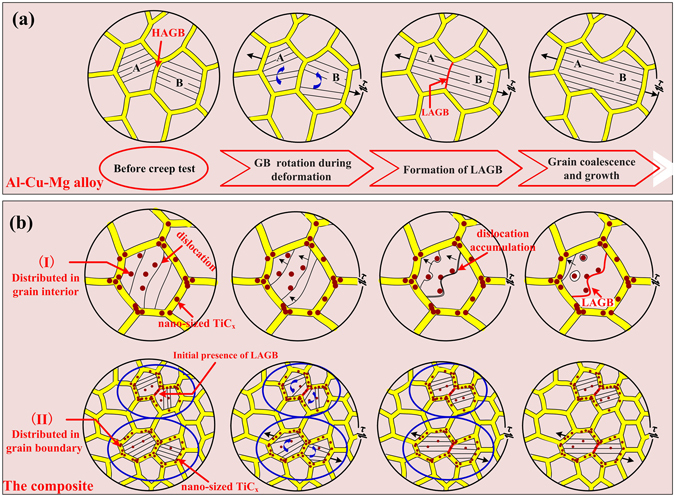
Proposed schematic for deformation model of (**a**) Al-Cu-Mg alloy and (**b**) its composite.

It has been well established that the grain growth and plasticity of metallic materials often involve grain boundary sliding/migration and grain rotation^27, 28^. Generally, under effective interaction of high temperature and applied stress, grain rotation and grain boundary migration can be easily initiated, resulting in grain coalescence^29–32^. To better understand grain growth during the creep test, a proposed schematic of deformation model is provided in Fig. 8. Figure 8a exhibits the grain growth evolution in Al-Cu-Mg alloy and it can be viewed that grain A and B share a common HAGB before creep test. With a long time creep test, grain A and B rotate towards one-another until the HAGB between the two grains translates to LAGB. The LAGB subsequently vanishes due to its migration induced by the applied stress^33^, and leading to grain growth. Figure 8b illustrates schematic that how nano-sized TiC_x_ particles affect the microstructure of the present composite. It can be observed that nano-sized TiC_x_ particles distribute in both grain interiors and at grain boundaries (Fig. 1d), which can be depicted in model (I) and (II). In model (I), according to Orowan strengthening mechanism, a portion of dislocations mobility can be hindered by nano-sized TiC_x_ particles, resulting in the dislocations pile-up in the grain interior. The collective dislocations’ motion arrays that makes up LAGBs. Moreover, the other part of dislocations slip or climb through the nano-sized TiC_x_ and finally vanishes at grain boundaries. In model (II), nano-sized TiC_x_ are located at HAGBs and LAGBs. During the creep test, HAGBs will translate to LAGBs on account of grain rotation, which is semblable to the model mentioned in Fig. 8a. But the difference is that the translated LAGBs will be pinned by nano-sized TiC_x_ through Zener pinning^34^. Zener pinning plays a major role at boundaries, when the ratio for volume fraction of particle to its diameter (Fv/d) in alloys is larger than 100 nm^−1^. In addition, the already existed LAGBs will also be pinned. It might explain partially why the fraction of LAGBs in the composite increases after creep deformation (Fig. 6e and f).

**Figure 9:**
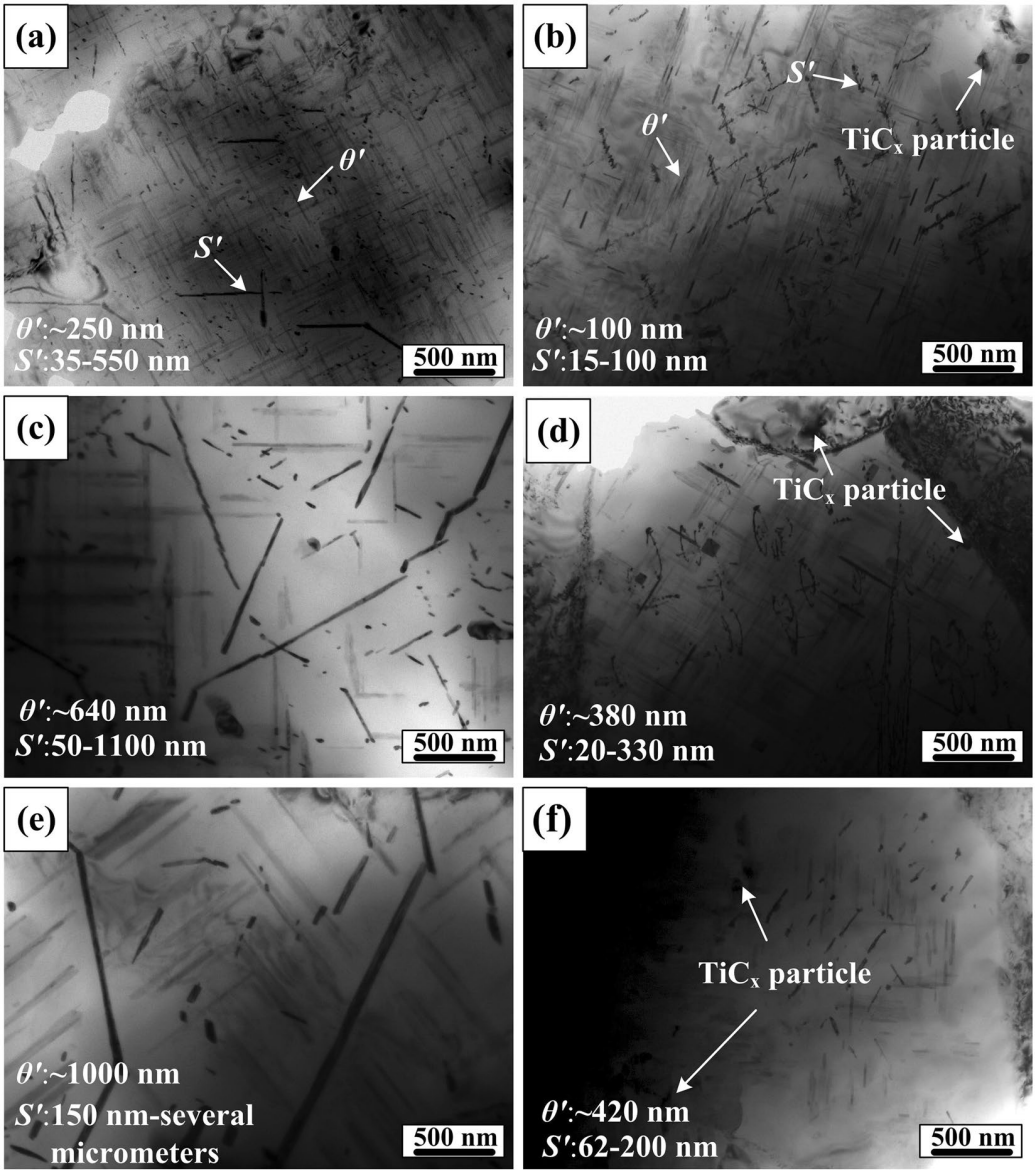
TEM images of *θ*′ and *S*′ precipitates in Al-Cu-Mg alloy and nano-sized TiC_x_/Al-Cu-Mg composite at various condition after tensile test: the (**a**) Al-Cu-Mg alloy and (**b**) the composite at 493 K, the (**c**) Al-Cu-Mg alloy and (**d**) the composite at 533 K as well as the (**e**) Al-Cu-Mg alloy and (**f**) the composite at 573 K.

Figure 9 shows TEM images of Al-Cu-Mg alloy and the composite having 9 vol.% nano-sized TiC_x_ after the creep test at 493 K, 533 K and 573 K under a stress of 60 MPa. All the pictures are taken along [001]_Al_ direction of α-Al. Apparently, the average sizes of *θ*′ and *S*′ precipitates in both Al-Cu-Mg alloy and the composite increase with the increase in temperature. After the creep test at 493 K, *θ*′ precipitates in Al-Cu-Mg alloy have an average size of about 250 nm, while the mean size of that in nano-sized TiC_x_/Al-Cu-Mg composite is merely about 100 nm, as shown in Fig. 9a and b. Moreover, the size of *S*′ precipitates is found to be inhomogeneous. At temperatures in the range of 533K-573K, the average size of *θ*′ precipitates in Al-Cu-Mg alloy grows from about 640 nm to 1 µm, while that of *S*′ precipitates increases from dozens of nanometers to several micrometers (Fig. 9c and e). Comparatively, both *θ*′ precipitates and *S*′ precipitates in the composite increase much more slowly in size (mainly below 500 nm, as shown in Fig. 9d and f). In addition, *S*′ precipitates of various shapes are observed in the composite. It is mainly due to the dislocations motion. It has been well confirmed that morphology, density and distribution characteristics of dislocations controls the shape, size and number density as well as distribution of *S*′ precipitates^21, 35^. During the creep test, climb and slip of dislocations are hindered by nano-sized TiC_x_ particles or *θ*′ and *S*′ precipitates, where *S*′ precipitates tend to precipitate group.

Nano-sized TiC_x_/Al-Cu-Mg composite exhibits high creep resistance compared with Al-Cu-Mg alloy at elevated temperatures, which can be explained via three aspects. The first one is particles strengthening by nano-sized TiC_x_. Fine-sized TiC_x_ particles, due to its thermal stability, can effectively pin the dislocation climb motion. Secondly, nano-sized TiC_x_ particles resist the movement of grain boundaries^36^, especially the LAGBs, during high temperature deformation. Thirdly, *θ*′ and *S*′ precipitates strengthening usually play significant roles in enhancing creep resistance of nano-sized TiC_x_/Al-Cu-Mg composite. Smaller size and more number of precipitates are more helpful for impeding the dislocations motion^22, 37^. However, *θ*′ and *S*′ precipitates will grow up and coarsen with further increase in temperature, which will result in the decrease of precipitates strengthening effect^20^. At last, nano-sized TiC_x_ particles, *θ*′ and *S*′ precipitates in the matrix prevent dislocations from moving, while pinning effect of nano-sized TiC_x_ particles keep grain boundary from sliding and rotation, resulting in higher creep resistance for the composites containing nano-sized TiC_x_ particles.

It can be concluded from the study that the creep resistance ability of nano-sized TiC_x_/Al-Cu-Mg composite was about 4–15 times higher than those of its matrix alloy under the similar testing conditions. The apparent activation energies for the composite were found to be 215, 210, 204 and 200 kJ/mol under applied stresses of 40, 60, 80, 100 MPa, respectively, which were much higher than those (177, 163, 155 and 150 kJ/mol, respectively) of Al-Cu-Mg alloy. True stress exponent of 5 for both composite and its matrix indicated that their creep behaviors were controlled by dislocation climb. The improved creep resistance of the composite were attributed to Orowan strengthen and pinning of grain boundary by nano-sized TiC_x_ particles as well as *θ*′, *S*′ precipitates.

## Methods

In our work, carbon nanotubes (CNTs, 10 nm-20 nm in diameter and 20 µm-100 µm in length), Ti powders (~48 µm) and Al-Cu-Mg alloy powders (~75 µm, containing 3.7 wt.% Cu, 1.3 wt.% Mg, 0.25 wt.% Si and 0.05 wt.% Fe) were used. First, Al-Cu-Mg alloy powders were mixed with Ti and CNTs powders (molar ratio of 1:1) at a speed of 100 rpm for 48 h, and then the blended powders were compressed into the cylindrical compacts with dimension of Φ45 × 35 mm. Subsequently, the cylindrical compacts were reacted in a self-made vacuum furnace and the temperature in the furnace was measured by the W5-Re26 thermocouple. The temperature measured suddenly rose rapidly, indicating that combustion synthesis reaction should be occurred and then quickly pressed the reactants while they were still soft. Finally, samples for the 9 vol.% nano-sized TiC_x_/Al-Cu-Mg composite were obtained when reactants cooled down to the room temperature. Besides, Al-Cu-Mg alloy was prepared under identical conditions.

An extrusion ratio of 19:1 for both the present composite and its matrix alloy were used during hot extruded process at 773 K. The extruded samples were then solution-treated at 783 K for 60 min, water quenching and followed by aging at room temperature for 96 h (T4 treatment). The creep tests were conducted on a creep-testing apparatus (RDL50, Changchun, China) under applied stresses of 40, 60, 80 and 100 MPa at 493 K, 533 K and 573 K, respectively. Transmission electron microscopy (TEM, JEM 2100 F, Tokyo, Japan) and scanning electron microscopy (SEM, Tescan vega3 XM, Czech Republic) equipped with an Oxford NordlysMax EBSD detector were carried out to observe microstructure evolution. Step sizes of 2 μm and 1 μm were used for Al-Cu-Mg alloy and it composite, respectively.

### Data Availability

The datasets generated during and/or analysed during the current study are available from the corresponding author on reasonable request.
